# Ice-Templated W-Cu Composites with High Anisotropy

**DOI:** 10.1038/s41598-018-36604-9

**Published:** 2019-01-24

**Authors:** André Röthlisberger, Sandra Häberli, Fabio Krogh, Henning Galinski, David C. Dunand, Ralph Spolenak

**Affiliations:** 1 Laboratory for Nanometallurgy, Department of Materials, ETH Zurich, Vladimir-Prelog-Weg 1-5/10, CH-8093 Zürich, Switzerland; 2 Mechanical Integrity of Energy Systems, Swiss Federal Laboratories for Materials Science and Technology, EMPA, CH-8600 Dübendorf, Switzerland; 3 Department of Materials Science and Engineering, Northwestern University, Evanston, IL 60208 USA; 4 Present Address: BIOTRONIK, Bülach, Switzerland; 5 Present Address: AXETRIS, Kägiswil, Switzerland

## Abstract

Controlling anisotropy in self-assembled structures enables engineering of materials with highly directional response. Here, we harness the anisotropic growth of ice walls in a thermal gradient to assemble an anisotropic refractory metal structure, which is then infiltrated with Cu to make a composite. Using experiments and simulations, we demonstrate on the specific example of tungsten-copper composites the effect of anisotropy on the electrical and mechanical properties. The measured strength and resistivity are compared to isotropic tungsten-copper composites fabricated by standard powder metallurgical methods. Our results have the potential to fuel the development of more efficient materials, used in electrical power grids and solar-thermal energy conversion systems. The method presented here can be used with a variety of refractory metals and ceramics, which fosters the opportunity to design and functionalize a vast class of new anisotropic load-bearing hybrid metal composites with highly directional properties.

## Introduction

The number of materials performing well in extreme environments, such as high temperatures above 2000 °C, high voltage or hazardous radiation fields, is limited and typically comprises high-melting materials, for example oxides^1^ and metal alloys based on niobium (Nb), molybdenum (Mo), tantalum (Ta), tungsten (W) and rhenium (Re). These metals, commonly dubbed “refractory metals” and their alloys find wide application in nuclear reactors^2,3^, turbines^3^, space-crafts^3^, thermal emitters^4^, heat spreaders^3,5^ and circuit breakers^6^ in power grids. Moreover, refractory metals are attractive candidates for plasmonic^7,8^ or photonic^9–12^ materials serving in solar-thermal energy conversion systems. Nevertheless, there are still substantial limitations regarding the fabrication and processing of new refractory metal-composites. Due to the large melting point, fabrication requires high temperature annealing making the design of new materials extremely challenging.

Of particular interest is the assembly of functional composites with anisotropic phase orientations^13–18^ which would allow for a highly directional materials response, e.g. anisotropic thermal or electrical conductivity. The fabrication of refractory metal based composites is typically restricted to powder metallurgical techniques. These powder-based methods have significant shortcomings because the materials produced are limited to isotropic composite structures with limited access to their properties and unavoidable residual porosity.

Here, we report on a different approach to create refractory metal-based composites utilizing ice-templating^19,20^ in a thermal gradient to assemble an anisotropic refractory metal scaffold which is then infiltrated by a second liquid phase. Ice-templating or freeze-casting is a remarkable process, using the anisotropic growth kinetics of ice during freezing to assemble suspended particles in a lamellar microstructure. The thickness of lamellae within the composite can be controlled by altering the velocity of the freezing front, while their volume fractions is given by the particle concentration in the suspension. This method has been successfully applied to fabricate various of complex composites, including synthetic nacre^21^, bio-materials^22^, porous ceramics^23^, ceramic-polymer composites^18^ and metals^24–29^. Compared to other techniques such as conventional extrusion, ice-templating has several advantages ranging from control of the macropore size to its sustainability as water is used instead of non-aqueous or polymeric templates that need to be burned out^20^. We have selected W-Cu composites as specific example, as W-Cu composites offer unique heat- and electrical transport properties paired with excellent mechanical strength at ambient and elevated temperatures, due to the outstanding thermal and electrical conductivity of copper combined with the high strength of tungsten. Historically, W-Cu composites proved to be an ideal testbed to understand fibre-reinforcement in composites^30^. These findings are still seminal for the understanding and functionalization of bio-inspired materials^31^, such as platelet reinforced polymers^32,33^ or artificial nacre^34,35^. W-Cu composites excel in applications where improved heat and current transport properties under extreme mechanical or thermal conditions are needed^36,37^ including electrodes for electrical discharge machining^38,39^, arcing contacts in high voltage circuit breakers^6,40^, fusion energy applications^3,5^ or heat spreaders with low coefficient of thermal expansion^3,41^.

**Figure 1:**
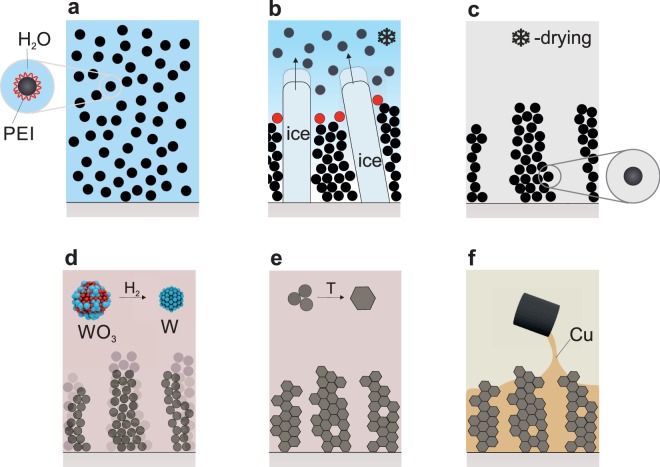
Schematic of the ice-templating process to create the anisotropic tungsten lamellar scaffold for melt-infiltration. The process steps involve (**a**) slurry formation by dispersing the ceramic powder (WO_3_) in water using PEI as surfactant, (**b**) formation of an anisotropic composite by ice-templating, particles (red) are captured between the ice-walls, i.e. lamellar dendrites (**c**) sublimation of ice to yield a loosely-bound WO_3_ scaffold with directional porosity, (**d**) reduction of the (WO_3_) scaffold yielding a metallic tungsten scaffold and (**e**) sintering of the porous tungsten. Significant volume shrinkage is associated with densification upon reduction (ceramic to metal) and sintering (closing of interparticle porosity). (**f**) Melt infiltration of the tungsten scaffold with liquid copper followed by solidification.

We fabricated anisotropic W-Cu composites using a three-step process illustrated in Fig. 1(a–f), starting with ice-templating a tungsten oxide (WO_3_) precursor suspended in water (Fig. 1(a)). Directional solidification is used to direct the assembly of a highly directional frozen green body, that is reduced and sintered to a metal scaffold by annealing (Fig. 1(c–e)). In a final step, this lamellar metallic scaffold is infiltrated by liquid copper and solidified, as shown in Fig. 1(f). Detailed information on the synthesis and processing of the tungsten skeleton is given in a previous work^25^.

To investigate the role of anisotropy in this composite, the microstructural, electrical and mechanical properties of the ice-templated W-Cu composite were measured and compared to commercially available isotropic W-Cu composites fabricated by powder metallurgy^42^. High-resolution x-ray computed tomography (XCT) provided three-dimensional information about the microstructure and anisotropy, four-point probe resistivity measurement gave insight on the impact of anisotropy on the electrical properties and uniaxial compression test were used to analyze the mechanical response of the composites. The results are discussed in the following.

## Results and Discussion

### Composite Architecture

**Figure 2:**
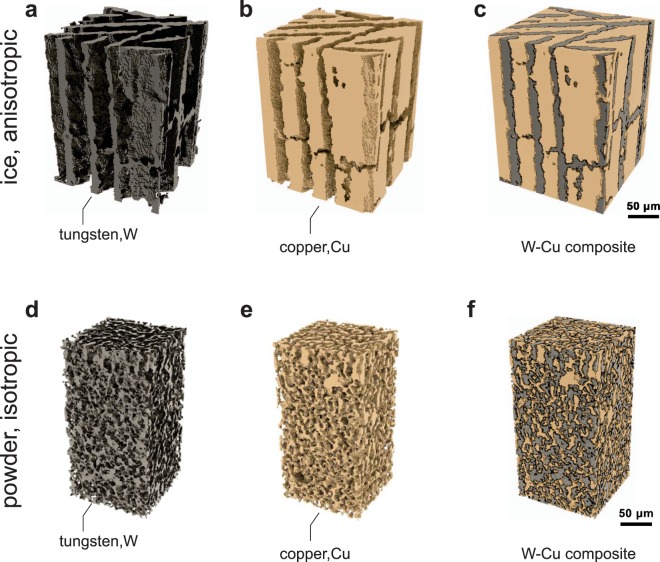
Composite Architecture. Three-dimensional X-ray computed tomographic reconstructions of W-Cu composites. (**a**–**c**) Show the Cu (**a**) and W (**b**) phases of an anisotropic lamellar composite produced by freeze-casting and reduction of a WO_3_ precursor, sintering and Cu infiltration; the volume fraction of W is 57% W/Cu. Panels (**d–f**) show the different phases of an isotropic W-Cu composite (65 vol.% W) produced by partial sintering of W powders followed by Cu infiltration. For both reconstructions the voxel size is 1.3 *μ*m.

To image the microstructure of the ice-templated and powder metallurgy based W-Cu composites, we resort to high resolution x-ray computed tomography (XCT). While allowing for a sub-micron resolution, XCT is a widely used, non-destructive technique to characterize complex materials and composites^43–46^. Figure 2 shows reconstructed three-dimensional images, including the composites individual phases, of the two analyzed W-Cu composites. The composite fabricated by ice-templating shown in Fig. 2(a–c) exhibits a lamellar architecture with a high structural anisotropy. The tungsten skeleton has been fully infiltrated by copper, resulting in an ordered composite with a volume fraction of 57% for W and 43% for Cu. These findings are supported by optical micrographs of radial and longitudinal cross-sections (see Supplementary Information, SFig. 3).

Scanning electron micrographs taken from a longitudinal cross-sections (see Supplementary Information, SFig. 2) revealed good wetting of Cu on the W-scaffold and an average grain size of 169 nm for Cu and 200 nm for W. In order to determine remaining porosity in the composite He-pycnometry measurements were performed, revealing 5 vol.% of closed porosity arising from the tungsten foam synthesis during freeze-casting. To quantify the anisotropy, i.e. determine the spatial periodicity and orientation of the lamellae within the composite, we used three-dimensional fast Fourier transform (3D FFT), detailed information is given in the Supplementary Information (SFig. 1). Two sets of lamellae rotated 40° relative to each other with a mean spatial frequency of 34 ± 2 *μ*m have been identified. Quite interestingly, the height of the ice-walls is generally not constraint in growth direction as long as the longitudinal thermal gradient is maintained. Accordingly, ice-templated composites can be fabricated, where the aspect ratio, i.e. height/width of each lamella is much larger than achieved here. This situation resembles platelet reinforced composites^32,47^, where the aspect ratio of the platelets critically impacts the mechanical properties.

Conversely, when powder metallurgical methods are used, the composite as shown in Fig. 2(d–f) is highly isotropic and characterized by two intermingled percolating phases. The structural analysis by 3D FFT (SFig. 1 Supplementary Information) reflects this result showing no directionality and reveals a spacing between the Cu and W phases of 9 *μ*m. In both cases, the spatial frequency of the composite is defined during the tungsten scaffold formation. For the powder metallurgical process, the powder size as well as the pressure and temperature during hot pressing determine the size, fraction and tortuousity of the porosity and thus the overall composite morphology after melt-infiltration with copper.

In the case of ice-templating, the scaffold architecture (W lamella thickness, channel width and orientation) is defined by the fraction of powder in the slurry: the W lamella thickness increased from 28 to 49 *μ*m with increasing the slurry particle fraction from 30 to 35 vol.% (Table 1). Also important is the solidification velocity, which controls the dendrite spacing and the extent of pushing and accumulation of the ceramic nanoparticles between the growing ice dendrites^19,23,48,49^. Finally, the sintering temperature and time (and the use of sintering aid) control the porosity within the walls, as well as the extent of shrinkage of the scaffold and thus the final W wall and channel width.

**Table 1:** Mechanical and structural properties including: volume fraction *f*_Cu_, lamella thickness *W*, Young’s modulus *E*, compressive strength *σ*.

	*f*_Cu_ [vol.%]	*W* [*μ*m]	*E* [GPa]	*σ* [MPa]
ice	27	49	266	1236
ice	35	28	259	1125
ice	38		255	983
ice	43		222	904
ice	48		237	866
ice	66		211	691
powder	35		234	1136
Cu	100		111	290

It is to note, that the scattering of the Young’s modulus data is attributed to measuring during loading, as the composites were tested without load-unload loops.

### Electrical Properties

**Figure 3:**
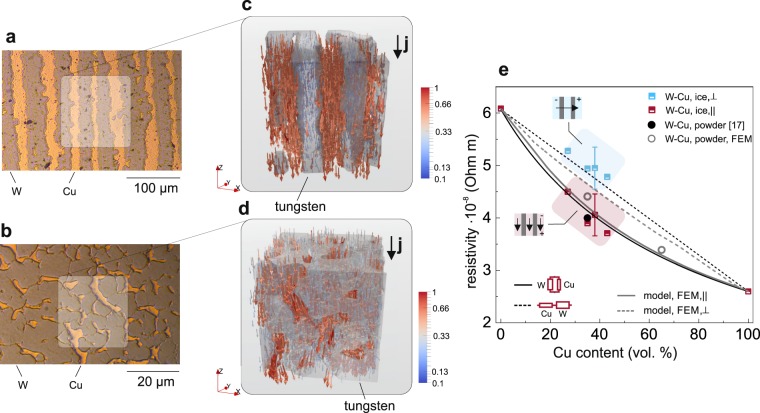
Electronic Transport. Panels (**a** and **b**) show polished cross-sections of ice-templated and powder-metallurgy templated W-Cu acquired using light microscopy. The electronic transport properties are modelled using FEM on a subset of the measured XCT data. Panels (**c** and **d**) present the simulated local current flow (arrows) and normalized current density distribution (arrow colour) within an ice-templated (**c**) and powder-metallurgy (**d**) W-Cu composite. The high resistivity regions, i.e. the W-phase (grey), correlate with regions of low current flow. (**e**) Comparison of the anisotropic, i.e. ice-templated, composite resistivity observed in experiments with theoretical predictions based on the FEM model illustrated in panels (**c** and **d**) and a parallel (${[\varphi /{\rho }_{{\rm{Cu}}}+(1-\varphi )/{\rho }_{{\rm{W}}}]}^{-1}$) or linear (*ϕρ*_Cu_ + (1 − *ϕ*)*ρ*_W_) combination of two resistors. Here, *ϕ* is the volume fraction of the Cu-phase and *ρ* the resistivity. In addition, the resistivity of the used powder based composite taken from ref.^42^ (black circle) is compared to the resistivity determined by the FEM model for a powder-based structure (open-grey circles).

Using the experimental three-dimensional XCT data, we can build representative finite-element-method (FEM) models to study the electronic transport properties of the fabricated W-Cu composites. Of special interest is the impact of structural anisotropy on the composites resistivity. When current or heat is transported through a lamellar structure, one can imagine two distinct situations, namely the flow of current parallel to the lamellae and across the lamellae. In both cases the local current density j(r) is given by Ohm’s law j(r) = *ρ*(r)^−1^E(r), where E(r) is the electric field at a given location r and *ρ* is the local, material-dependent resistivity. In our samples (Fig. 3(c,d)), due to the resistivity contrast between the W ($6.0\pm 0.4\cdot {10}^{-8}$ Ωm) and Cu-phase ($2.6\pm 0.4\cdot {10}^{-8}$ Ωm), the current is predominately transported within the Cu phase as shown by FEM simulations for ice-templated and powder-based composites, respectively. In both composites, the current density within the Cu-phase is enhanced by up-to one order of magnitude, see (Fig. 3(c–e)). To this extend, we can introduce an effective resistivity that treats the current transport in the composite within the bounds of two parallel resistors for the lower limit and two resistors in series for the upper limit. Thereby the resistivity of each resistor corresponds to the pure Cu and W phase, respectively. By applying experimental values for these two parameters, we can calculate the resistivity as function of Cu content and model the resistivity by solving Ohm’s law within the FEM model. A detailed description of the FEM model is given in the in the Supplementary Information). In Fig. 3(e) the experimentally determined resistivity values are compared with these theoretical predictions.

The electrical resistivity of the composites, bulk copper, and bulk tungsten were measured by using the four-point probe method: for the pure metals values of $2.6\pm 0.4\cdot {10}^{-8}$ Ωm for Cu and $6.0\pm 0.4\cdot {10}^{-8}$ Ωm for W were found. The determined values agree with values from literature^50,51^. Using a linear array probe head enables a directional measurement of the resistivity parallel and perpendicular to the lamellae in the ice-templated structure (see also Supplementary Information SFig. 5). The corresponding resistivity values are reported and highlighted in Fig. 3(e) by the light blue and pink areas.

The FEM simulations and the circuit models reproduce well the experimental results, confirming the anisotropic electronic transport properties of the lamellar composite. The lamellar structure of the composite, introduces a directional dependence of the current flow, which results in anisotropic electrical resistance as shown in Fig. 3(e). As expected, the powder-based composite exhibits isotropic behaviour (Fig. 3(e), light grey circles), while the overall resistivity exceeds the parallel resistor circuit. The FEM simulation indicate that the current transport in the isotropic composite is confined to the Cu-phase, see Fig. 3(d), whose tortuosity controls the composite resistivity. A tortuous path effectively increases the conductive path and hence the resistivity of the composite, as compared to the parallel resistor model without tortuosity.

It is noteworthy, that similar effects, due to the analogy of Ohm’s law and Fourier’s law, can be expected for heat conduction given a significant difference in thermal conductivity of both materials^52^.

### Mechanical Properties

**Figure 4:**
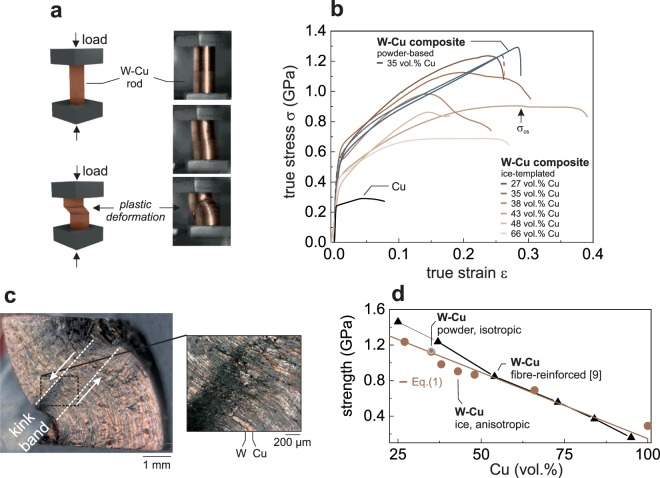
Mechanical behavior. Compressive properties of ice-templated and powder-metallurgy W-Cu composites under compression. Panel (**a**) shows a schematic illustration and photographs of the compressive tests of an ice-templated W-Cu composite with 43 vol.% Cu. (**b**) Experimental stress-strain curves of W-Cu composites with various Cu volume fractions. All ice-templated composites, measured with lamellae parallel to the applied stress, feature a region of plastic instability after the yield strength is reached. The compressive strength is labelled *σ*_cs_ as an example for a composite with 43 vol.% Cu. (**c**) Optical micrograph image of an ice-templated W-Cu composite (66 vol.% Cu) after compressive testing affirming kink band formation as failure mode. The zoomed-in view highlights the nearly uniform misorientation of the composite within the kink band. Panel (**d**) compares the measured compressive strength of (Fig. 4)**b** with theoretical predictions based on a simple rule of mixture (Eq. 1) and compressive strength measured for fibre-reinforced W-Cu composite tested in the fiber direction, from ref.^9^.

The mechanical properties of the composites are determined by uni-axial compression test (Fig. 4a), which is an ideal technique to characterize the elastic and plastic response of a material to an external load. In Fig. 4a the different stages of deformation - from elastic to plastic deformation to shearing are depicted by photographs taken during testing. The stress-strain curves in Fig. 4b show distinct behavior depending on the composites’ architecture. In both cases, no sharp yield point is visible but rather a gradual transition from the elastic to the plastic regime is observed. The powder-based isotropic composite exhibits a classical transition from linear elastic to linear plastic deformation terminated by brittle fracture once the compressive strength is reached. By contrast, the ice-templated composites feature a different mechanical response. The stress-strain curves of the anisotropic composite exhibits next to its linear elastic region a nonlinear plastic region including softening after the compressive strength of the material is reached. The measured mechanical properties, such as compressive strength and Young’s modulus are summarized in Table 1.

The origin of strain softening can be explained by considering the interaction between the strong, brittle phase (W) and the weak, ductile phase (Cu) in the composite, whose yield stress inducing plastic flow is much smaller than the failure stress of the former. In such a situation, the plastically flowing Cu phase interacts with elastically deformed W-lamellae via shear forces at the Cu/W interface. For such a composite made of strong fibers within a plastically-deforming matrix, Kelly and Tyson^30^ have introduced a linear rule of mixture that captures these two coupled deformation mechanisms. This rule of mixture, suited for platelet-based composites as well^53^, superimposes the compressive strength *σ*_*cs*,*i*_ of the single components to predict the effective compressive strength *σ*, leading to:

1 $$\sigma =\alpha \cdot {\sigma }_{{\rm{cs}},{\rm{W}}}\,(1-\varphi )+{\sigma }_{{\rm{cs}},{\rm{Cu}}}\varphi .$$

Here, *ϕ* is the volume fraction of the Cu-phase and $\alpha =1-\tfrac{{\sigma }_{cs,{\rm{W}}}t}{{\tau }_{y,Cu}L}$ is the reinforcement factor containing the ratio between the compressive strength of tungsten *σ*_*cs*,*W*_ and the shear strength of copper *τ*_*y*,Cu_ and the ration between the lamellae thickness *t* and the lamella length *L*.

We validate the model against the experimental results, using values *σ*_cs,W_ = 1686 MPa, *σ*_cs,Cu_ = 147 MPa. and *α* = 0.96 with values for *σ*_*cs*,*W*_ and *τ*_*y*,Cu_ from refs^54^ and^55^. Figure 4d reports the calculate compressive strength using Eq. 1 and the experimentally measured compressive strength as function of Cu content. The linear decrease in strength predicted by Eq. 1 is well represented in our experimental data, although some deviation is found for intermediate Cu-contents. The good agreement with the rule of mixture originates from the fact that the elastic and plastic deformation until the compressive strength is reached seem only slightly affected by the composite structure. In the elastic regime, the tungsten skeleton is carrying a majority of the load, as copper yields below 50 MPa. Since tungsten is very brittle at room temperature, little to no plastic behavior of the tungsten skeleton is expected^3^. Therefore the observed ductility of the composite until compressive strength is attributed to the plastic deformation of the copper matrix, despite the presence of a continuous tungsten phase^56^. The compressive strength rises with decreasing copper content (Fig. 4d) in the ice-templated composites, due to much higher strength and stiffness of the tungsten phase. Especially, for a Cu-content <50%, the strength of ice-template composites is significantly smaller than the strength of fibre-reinforced composites. This reduction in strength can be attributed to the presence of additional 5 vol.% of closed porosity in the W scaffold. The effect of anisotropy on the mechanical response of the composites comes into play once the compressive strength (*σ*_*cs*_) is reached. Beyond this critical value the mechanical response is nonlinear as featured in the stress-strain curves and exhibits significant softening, see Fig. 4b. In contrast to the powder-based composites, the lamellar architecture of the ice-templated W-Cu structures offer no direct path for cracks to propagate perpendicular to the load axis suppressing brittle fracture at *σ*_*cs*_. Rather, it is expected that the applied compressive stress will produce a local shear stress in regions where the W-lamellae are misaligned with respect to the compression direction. The initial shear and rotation in these regions is the onset for “kink band” formation, a deformed and rotated region within the compressed sample, which is a common deformation mode in anisotropic composites^57–59^, such as Cu-Nb nanolaminates^60,61^. This deformation mode is expected in our lamellar structure with mechanical anisotropy under compression. The strain softening observed in Fig. 4b and formation of a geometrical deformed band during compression, as shown in Fig. 4c and the Supplementary Information Video S1 and SFig. 6, are key characteristics for kink band formation. The formation of the kink band localizes the plastic deformation of the sample during compression as shown in Fig. 4c and Video 1, resulting in kink-band broadening^58^ with increasing strain.

## Conclusion

We have demonstrated a template-based approach to fabricate anisotropic refractory metal-based composites. Here, ice-walls grown in a thermal gradient have been used to create lamellar W-Cu composites with a highly anisotropic lamellar architecture. Using this technique lamellar composite architectures or laminates with an average lamella spacing of 34 *μ*m and lamella thickness between 28–49 *μ*m have been produced. We have studied the composites’ architecture including electronic and mechanical properties in direct comparison with a purely isotropic W-Cu composite. In particular, we observe a directional dependence of the composites resistivity, that can be engineered by changing the Cu content of the composite. Applying a combination of analytic and numerical techniques to the measured 3D XCT data, we demonstrate that the current density is enhanced by one order of magnitude within the Cu phase as compared to the W phase, while the overall composite resistivity can be explained in first approximation by a simple combination of two resistors in parallel or in series. Similar to fibre-reinforced composites, we demonstrate that strength of the lamellar composites follows a simple rule of mixture, whereby the anisotropy of the structure leads to nonlinear plastic behaviour and significant strain softening induced by kink band formation after the compressive strength is reached.

The refractory metal based lamellar composites introduced in this work open up new possibilities to design anisotropic, mechanically enhanced materials especially for energy applications. Ice-templating is rapid, simple and potentially scalable to large areas, and can produce tailored anisotropic composites for a new generation of circuit breakers, thermal emitters and large scale photonic and plasmonic components. Here W-Cu is used as a testbed, but the introduced concept is applicable to a wide range of refractory metals and lower-melting, high-ductility matrices such as Cu alloys, Ni-alloys and Co-alloys. Furthermore, the novel infiltration process of the metal scaffold presented is not limited to ordinary metals, but can be easily extended to polymer-melts, ceramic-slurries with sintering temperatures below the melting point of the metal scaffold, and even liquid electrolytes.

## Methods

### Composite Synthesis

Aligned tungsten scaffolds were created via hydrogen reduction and sintering of a green body, created by ice-templating of an aqueous slurry of tungsten trioxide nanoparticles (WO_3_, 99.95% purity, *d* < 100 nm, SkySpring Nanomaterials Inc.) using 0.5 wt.% nickel oxide (NiO, 99.9% purity, *d* = 20 nm, Inframat Advanced Materials), 2.5 wt.% polyethylene glycol (PEG, Mn = 3,400, Sigma-Aldrich) and 1 wt.% polyethylene imine (PEI, Mw 25,000, Sigma-Aldrich) as sintering activator, binder and dispersant respectively. The process is schematically summarized in Fig. 1. Here, a freeze-casting temperature of −20 °C has been used. The effects of processing parameters - powder fraction in the slurry, freeze-casting and sintering temperatures as well as the influence of nickel - on the final scaffold microstructure, in particular open porosity and structural wavelength, have been reported in detail in a previous publication^25^. The resulting anisotropic tungsten foam cylinders, were subsequently melt-infiltrated with Cu (99.99% purity, Mateck GmbH) at 1300 °C under Ar/5% H_2_ atmosphere (99.999% purity, PanGas). The copper was placed on top of the tungsten scaffold in an alumina crucible (99.8% purity Al_2_O_3_, 15 mm diameter, Metoxit GmbH) and melted by inductive heating. The temperature was monitored optically by a custom-built laser pyrometer. Upon melting, the copper infiltrates the tungsten foam due to gravitational forces. After solidification and cooling of the composites to room temperature, cylindrical compressive specimens (5–6 mm diameter and height/diameter aspect ratio of 2:1) were machined on a lathe. Control specimens with isotropic W distribution were obtained from W-20 wt.% Cu composites created via partial sintering of W powder preforms followed by Cu liquid infiltration (Plansee Powertech AG, Seon, Switzerland)^42^. These commercially-available specimens exhibit a grain size between 4–8 *μ*m, a density of 15.2 g/cm^3^ and a resistivity of $4\cdot {10}^{-8}$ Ωm.

### Microstructural, Electrical and Mechanical Characterization

Radial and longitudinal cross-sections (perpendicular and parallel to the slurry solidification direction, respectively) were cut and prepared according to standard metallography preparation and imaged via light microscopy. Broad ion beam (BIB) polished cross-sections of ice-templated composites have been analyzed using a scanning electron microscope (SEM, Zeiss Leo 1530). To compare the ice-templated and isotropic W-Cu composite microstructures, high energy (acceleration voltage 170 kV) X-Ray tomography (Phoenix Nanotom S, GE Measurements) was performed (voxel size 1.3 *μ*m) on 400 *μ*m diameter cylindrical composite samples machined with a lathe. The three-dimensional data where visualized using ImageJ and Blender, whereby Parallel FFTJ was been used to calculate the three dimensional fast Fourier transform (FFT) of the composites. To build the 3D FEM models the XCT-data have been used. Hereby, the 3D-surfaces of the different phases have been imported to Comsol Multiphysics. In case of lamellar composites, the volume fraction within the FEM model can been altered by anisotropically scaling the components of one of the two phases. A detailed illustration is given in the Supplementary Information (SFig. 4). Compression tests were conducted at 25 °C, well below the brittle-to-ductile-transition of tungsten reported^3^ as 280–330 °C, depending on strain rate^62^ and impurity level^63^. In all cases, the load axis was parallel to the lamellar structure. A screw-driven mechanical testing machine operated at constant crosshead displacement rate (corresponding to a sample strain rate of 10^−4^ s^−1^) was used, with sample strain measured optically using digital image correlation. Four-point resistivity measurements (National Instruments, NI PXI-4071 and Agilent, N6700B 400 W, for voltage measurement and current supply, respectively) were conducted in steady-state conditions with current flow parallel and perpendicular the lamellar structure to obtain the anisotropic electrical resistivity of the composites. For this purpose, a Jandel Multiheight probe station equipped with a Jandel linear four point probe head (tip spacing = 1 mm) was used. These measurements were performed on polished disk-shaped samples with a typical diameter of 15 mm and height of 3 mm.

## Electronic supplementary material

LaTeX Supplementary File

LaTeX Supplementary File

LaTeX Supplementary File

Supplementary Information

Video
