# Supplementary material for: Ice-Templated W-Cu Composites with High Anisotropy
Source: Sci Rep. 2019 Jan 24;9:476. doi: 10.1038/s41598-018-36604-9 (PMC6346048; doi:10.1038/s41598-018-36604-9)
Supplement: Supplementary file 4 — Supplementary Information [file 41598_2018_36604_MOESM4_ESM.pdf]

Supplementary Information for:
Ice-Templated W-Cu Composites with High Anisotropy

André Röthlisberger[1,2†♮], Sandra Häberli[1∠], Fabio Krogh[1], Henning Galinski[1†], David C. Dunand [3] & Ralph Spolenak[1]

[1]*Laboratory for Nanometallurgy, Department of Materials, ETH Zurich, Vladimir-Prelog-Weg 1-5/10, CH-8093 Zürich, Switzerland*

[2]*Mechanical Integrity of Energy Systems, Swiss Federal Laboratories for Materials Science and Technology, EMPA, CH-8600 Dübendorf, Switzerland*

[3]*Department of Materials Science and Engineering, Northwestern University, Evanston, IL 60208, USA*

[†] These authors contributed equally to this work

♮ currently at BIOTRONIK, Switzerland

∠ currently at ABB, Switzerland

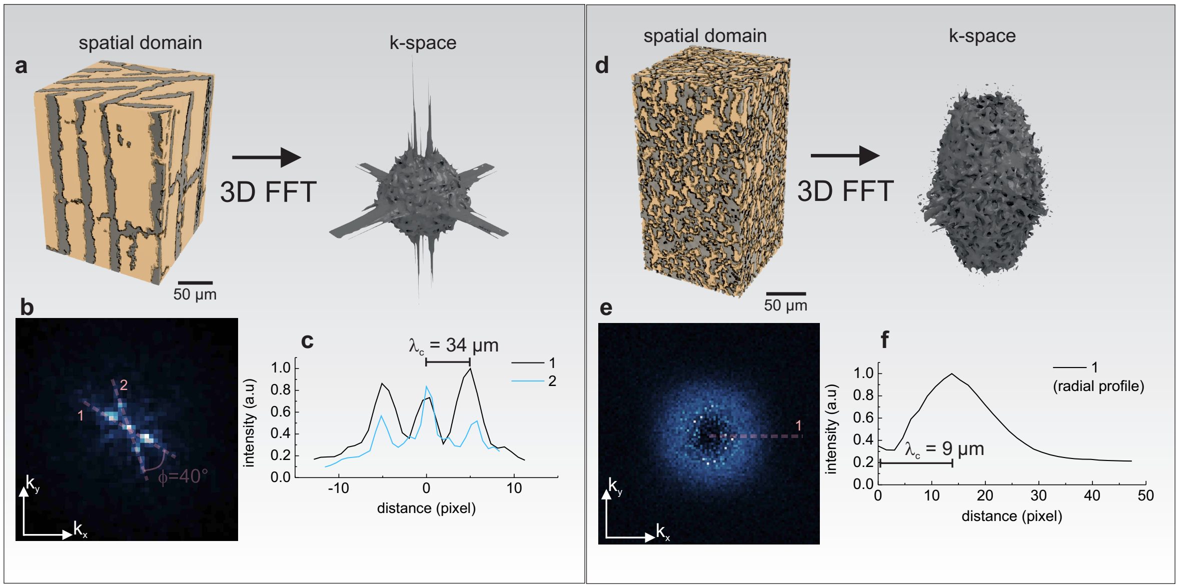

Figure S1 – FFT Analysis. (a) Three-dimensional Fast Fourier transformation (FFT) of the ice-templated W-Cu composite structure obtained by XCT using Parallel FFTJ and ImageJ. (b) x-y plane of the FFT, bright spots in k-space are indicating a clear spatial periodicity and structural anisotropy. Two features, i.e. two type of W-lamellae, with similar periodicity but rotated 40° relative to each other can be identified. (c) Line profiles along two directions (1,2) highlighted in (b) are shown. These profiles are used to measure the mean spatial frequency of the structure $34(2)$ μm. (d) Three-dimensional Fast Fourier transformation (FFT) of the powder-based W-Cu composite structure obtained by XCT using Parallel FFTJ and ImageJ. (e) x-y plane of the FFT, with a diffuse ring in k-space is indicating a highly isotropic structure with no distinct periodicity. (f) Line profile (1) highlighted in (e) is depicted, revealing a mean spacing between the Cu and W phases of 9 μm.

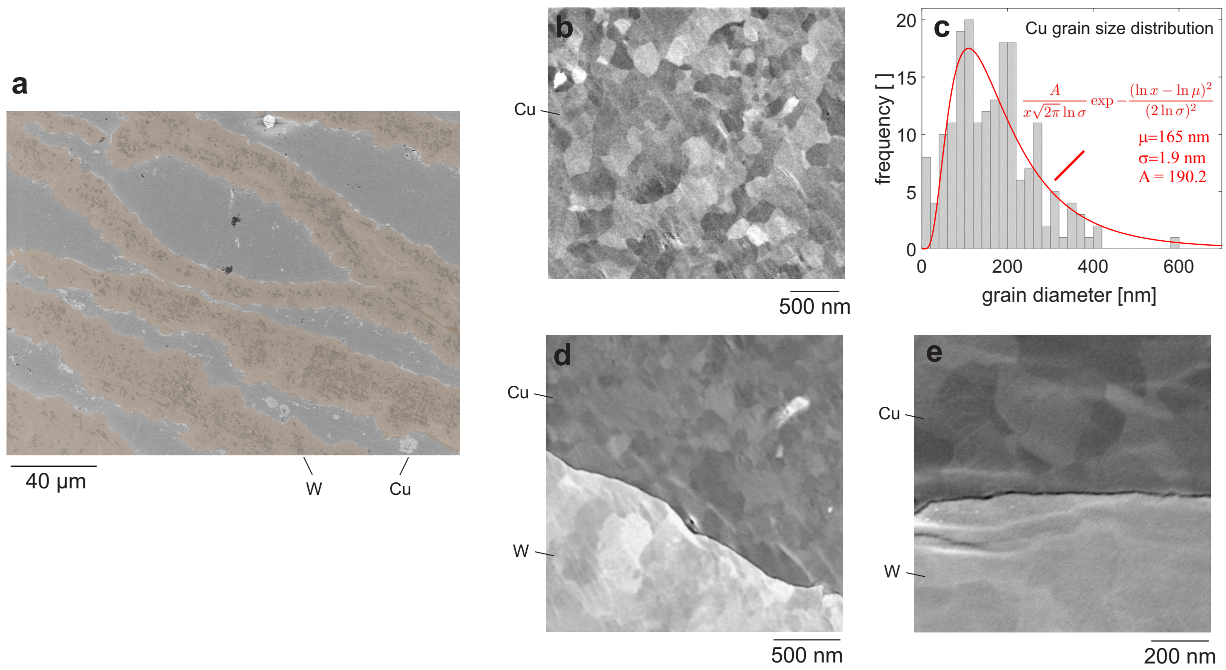

Figure S2 – Microstructure Analysis I. (a) False-colored scanning electron micrograph of a broad ion beam (BIB) polished longitudinal cross-section of a W-Cu composite (43 vol.% Cu) synthesized by freeze-casting. The W phase is colored brown. (b) Contrast-enhanced scanning electron micrograph highlighting the microstructure of the Cu phase. (c) The intercept method[1] is used to measure the grain size distribution in (b). The resulting Cu grain size distribution agrees well with a lognormal distribution with an average grain diameter of 165 nm. Panel (d)-(e), zoomed-in views on the W-Cu interfaces at two different magnifications and locations on the specimen, show excellent wetting between the tungsten phase and the melt-infiltrated copper. Furthermore, the W grain size has been measured based on panel (d) resulting in an average grain diameter of 200 nm. For both phases the average grain size is larger than 100 nm and, in this regime, classical deformation by dislocation-mediated plasticity is expected. As the electron mean free path (40 nm for Cu [2] and 16 nm for W [2]), is several times smaller than the grain size, the present microstructure will have no significant effect on the electrical conductivity.

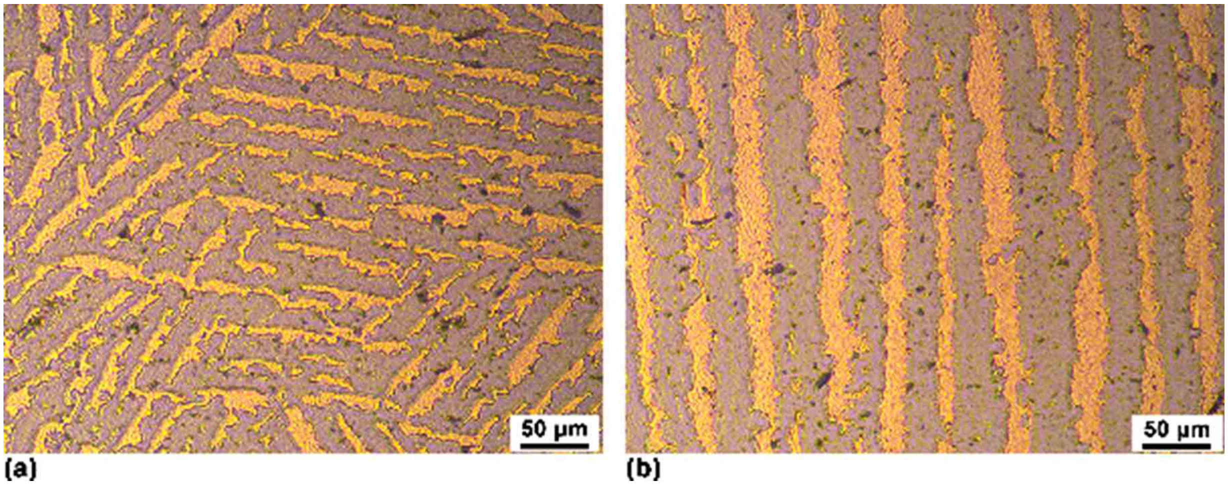

Figure S3 – **Microstructure Analysis II.** Radial (a) and longitudinal (b) metallographic cross-section of a W-Cu composite (43 vol.% Cu) synthesized by freeze-casting. The architecture is clearly anisotropic with the tungsten exhibiting the typical directional structure inherent to the freeze-cast foams and the copper filling the open porosity. The orientation of the walls is random in radial direction, while along the cylinder axis (longitudinal) it is parallel. The black spots are pores from the metallographic preparation. The shear forces created by the abrasive particles of SiC (as used in grinding) and diamond (used for polishing) are highest in the soft copper, removing slightly more copper and creating undulations.

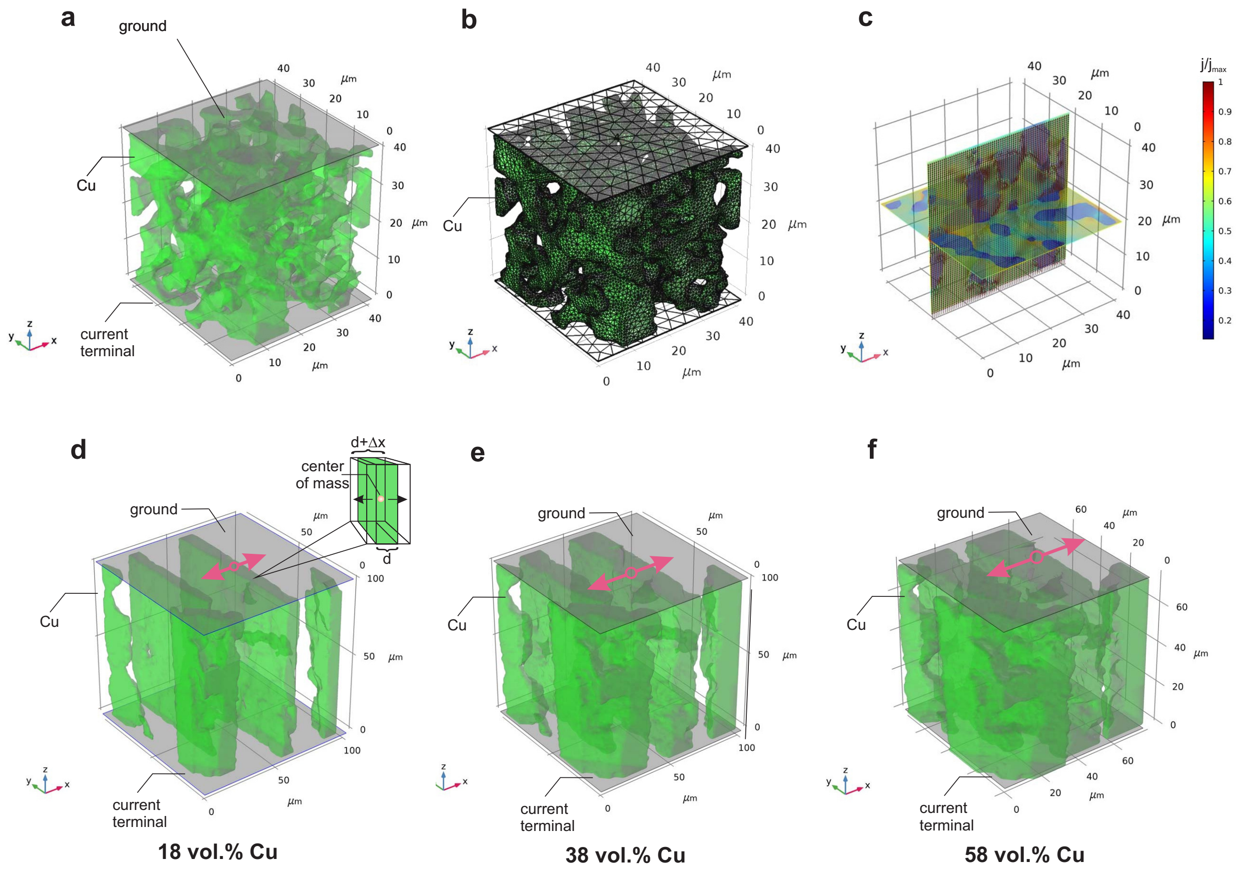

Figure S4 – Finite Element Modeling. (a) Illustration of the finite element model designed to determine the resistivity using the experimental three-dimensional XCT data. Here, a powder-based composite is used exemplary. Note, only the Cu-phase within the cube is shown. A ground and current terminal is created on the upper and lower faces perpendicular to the z-axis. All other faces are set to be electrically insulating. (b) Meshed finite element model created in (a). (c) Multi-slice colormap of the normalized current density within the composite. Using the geometrical factors, the current flow and the voltage difference the resistivity of the composite can be calculated. By placing the ground and terminal on faces perpendicular to the x or y-axis anisotropic composites can be modeled. (d)-(f) XCT data based finite element models of anisotropic ice-templated W-Cu composites with different Cu content, namely 18vol.% Cu (d), 38vol.% Cu (e) and 58vol.% Cu (f). To create composites with different Cu content, each Cu lamella within the XCT data set is anisotropically scaled with respect to its center of mass.

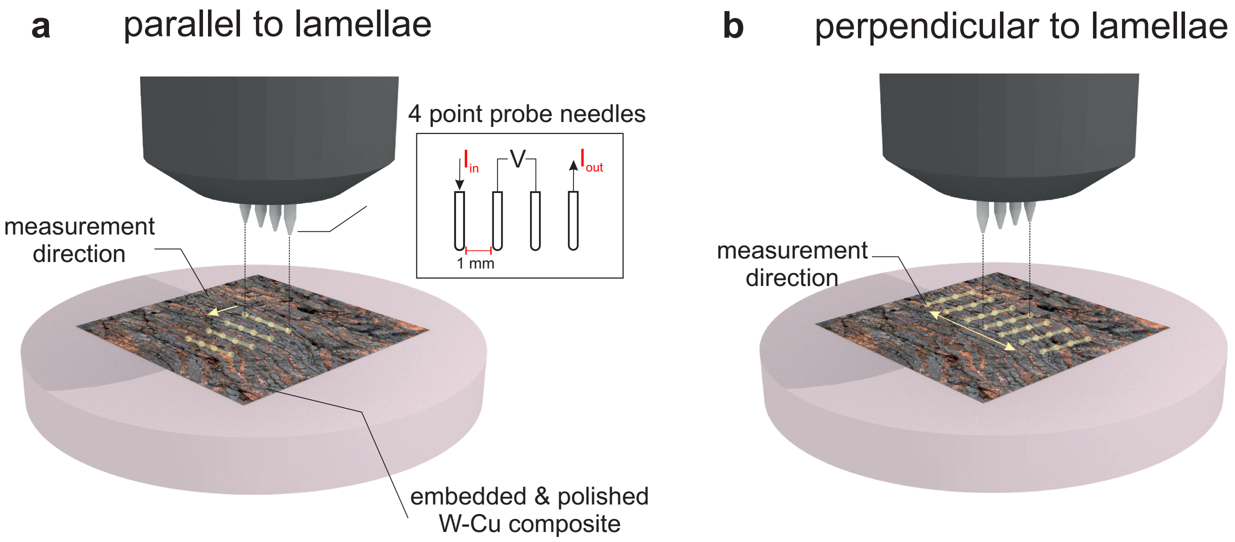

Figure S5 – 4 point probe resistivity measurements. The 4 point resistivity measurements have been performed using a 3.8 mm wide 4 point probe needle head. The samples have been embedded and polished prior measuring. The samples have been measured parallel (a) and perpendicular (b) to the orientation of the lamellae. On each sample the resistivity as been determined by averaging over several measurements from different locations.

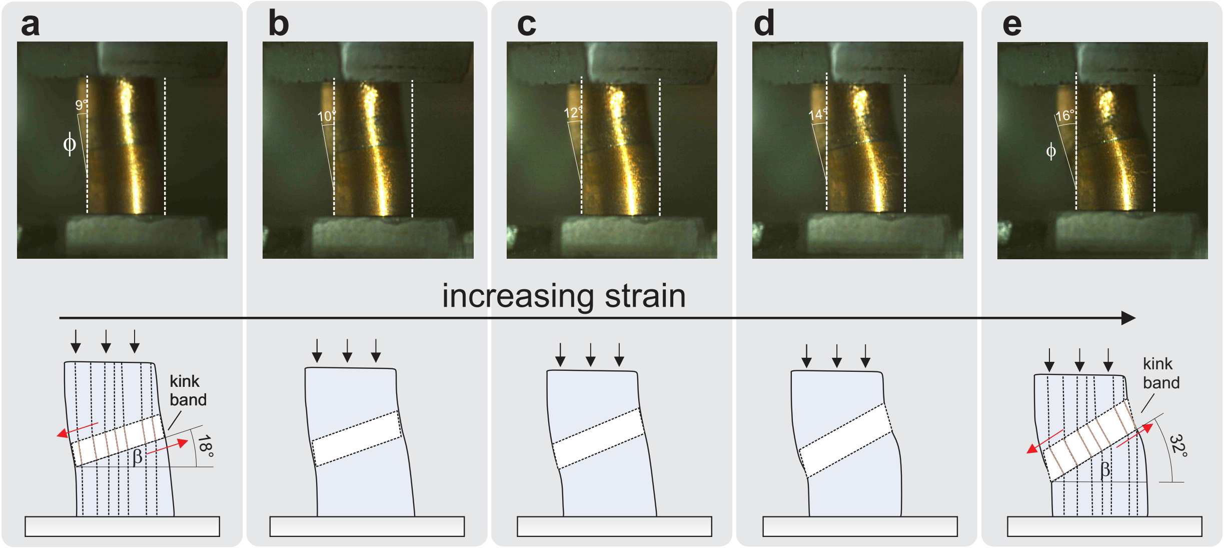

Figure S6 – Kink band propagation. Sequence of photographs (a)-(e) of an ice-templated W-Cu composite (43 vol.% Cu) during compressive testing illustrating kink band formation. The strain increases from left (a) to right (b). The outline of each sample is depicted together with the position and orientation of the kink band. For all states of compression, the rotation of the material, i.e. the angle of misorientation ϕ and the angle between the deformed and undeformed material β hold the relationship $\phi = 2\beta$, which is characteristic for kink band formation and propagation.

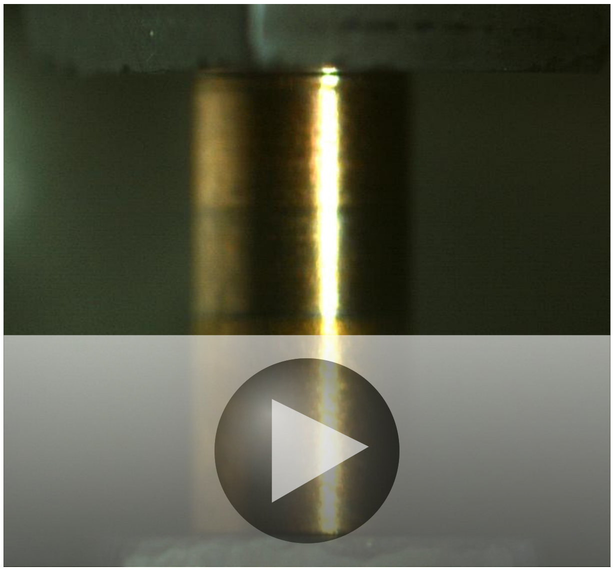

Video S1 – Compression Test. Video shows a compression test of an ice-templated W-Cu composite (43 vol.% Cu). During compression the sample exhibits kink band formation, i.e. a deformed and rotated band is formed within the sample.

1 References

1. Abrams, H. Grain size measurement by the intercept method. *Metallography* **4**, 59 – 78 (1971). URL http://www.sciencedirect.com/science/article/pii/002608007190005X. DOI https://doi.org/10.1016/0026-0800(71)90005-X.

2. Gall, D. Electron mean free path in elemental metals. *Journal of Applied Physics* **119**, 085101 (2016). URL http://dx.doi.org/10.1063/1.4942216. DOI 10.1063/1.4942216. http://dx.doi.org/10.1063/1.4942216.
